# Association Between Mental Health and Oral Health Status and Care Utilization

**DOI:** 10.3389/froh.2021.732882

**Published:** 2022-02-07

**Authors:** Tamanna Tiwari, Abigail Kelly, Cameron L. Randall, Eric Tranby, Julie Franstve-Hawley

**Affiliations:** ^1^School of Dental Medicine, University of Colorado Anschutz Medical Campus, Aurora, CO, United States; ^2^CareQuest Institute for Oral Health, Boston, MA, United States; ^3^Department of Oral Health Sciences, University of Washington School of Dentistry, Seattle, WA, United States

**Keywords:** mental health, oral health, stress, anxiety, psychology, values

## Abstract

Studies have shown that mental health and oral health may be correlated, with associations demonstrated between mental health problems and tooth loss, periodontal disease, and tooth decay. The COVID-19 pandemic had alarming implications for individuals' and communities' mental and emotional health. This study examined the associations between mental health status, oral health status, and oral healthcare utilization and highlighted the impact of COVID-19 on mental health. Additionally, this study examines specific sociodemographic factors that may amplify oral health disparities. A nationally representative survey was conducted to capture attitudes, experiences, and behaviors related to oral health, mental health, and unmet oral health needs. Eighteen percent of respondents were categorized as having poor mental health. Visiting the dentist in the last year was more common amongst individuals with good mental health. From the logistic regression model, mental health status, age group, race/ethnicity, education, and last dental visit were all significantly associated with of oral health status. Mental health status, age group, and income groups were all significantly associated with unmet oral health need. Future work should focus on the mental-oral health association, including determining ways to improve oral healthcare utilization and oral health status among people with poorer mental health.

## Introduction

Individuals with mental illnesses are more likely than those without to have suboptimal oral health [1]. Although the nature of this relationship has not been extensively evaluated, a few studies have shown a relationship between oral health and mental health, or vice versa [2–4].

One systematic review has addressed the association between poor oral health and common psychological disorders (e.g., a diagnosis of depression, generalized anxiety disorder, panic disorder, obsessive-compulsive disorder, post-traumatic stress disorder, or a phobia) [2]. The study demonstrated a significant association between common mental health disorders and tooth loss; individuals with common psychological disorders had higher rates of decayed, missing, and filled teeth surfaces than controls. Overall, the researchers concluded that the rates of dental decay and tooth loss were higher for patients with common mental disorders as compared to the general population [2]. Other studies have shown a relationship between poor oral health (e.g., chronic periodontitis, tooth erosion) and severe mental illness and eating disorders [3, 4].

In patients with depression and anxiety, past research documented the association between the use of oral health services and tooth loss [5]. Many depressive symptoms, such as anhedonia or lack of motivation, feelings of worthlessness, and fatigue, may adversely affect adults' behaviors related to oral hygiene maintenance [5]. Several studies demonstrated similar results related to depression, dental behaviors, and the management of dental diseases such as periodontal disease [6–8]. These studies highlight the public health implications of mental health on oral health outcomes and the potential cycle of relationship oral health has with mental health (and vice versa). Greater risk for dental decay and tooth loss can lead to more frequent pain experience, social isolation, and low self-esteem, and reducing quality of life and in turn possibly being associated with poorer mental and overall health [9].

The COVID-19 pandemic had alarming implications for individuals' and communities' mental and emotional health [10]. Factors including isolation and feelings of uncertainty negatively impacted mental health and included effects such as increased anxiety, loneliness, depression, insomnia, and in some cases, self-harm [10]. A large portion of the population experienced job loss and economic difficulties exacerbated mental health issues [11, 12].

The pandemic took a toll on oral health as well: dental practices were closed for a long period of time, elective dental procedures were postponed, and access to preventive care was delayed [13]. Many groups were disproportionately affected by the pandemic, including populations with a higher risk for poor oral health, those with other chronic diseases and comorbidities, low socioeconomic groups, and minority populations [14]. Although several studies reviewed the relationship between the pandemic and mental or oral health individually, none have examined the association between mental and oral health during the pandemic [11–14]. This study aimed to identify the associations between mental health, oral health, and oral healthcare utilization during the COVID-19 pandemic as well as specific sociodemographic factors that may amplify disparities.

## Methods

A nationally representative cross-sectional survey (*n* = 5,320) was conducted in January and February 2021 to assess consumer attitudes, experiences, and behaviors related to oral health. This study was deemed exempt by the WCG IRB.

The survey called the State of Oral Health Equity in America 2021 contained items related to oral health knowledge, dental care experiences, interprofessional care, insurance, and social determinants of health. The survey included ~150 questions. The questions were developed by an internal team at the CareQuest Institute comprised of knowledge experts on payer, provider, and patients' experiences with oral health.

The survey was administered to adults 18 years and older by the non-partisan research organization National Opinion Research Center (NORC) at the University of Chicago, using the AmeriSpeak panel. AmeriSpeak is a probability-based panel designed to be representative of the U.S. household population. Randomly selected U.S. households were sampled using area probability and address-based sampling, with a known, non-zero probability of selection from the NORC National Sample Frame. Sampled households were contacted by U.S. mail, telephone, and field interviewers. The survey was internally piloted at CareQuest Institute, as well as initially piloted by NORC on a sample size of 500. A sampling unit of 16,986 was used with a final sample size or weight sample was 5,320 with a final weighted cumulative response rate of 5.2%. All data presented account for appropriate sample weights. This sample size was decided in collaboration with NORC in order to obtain a margin of error of under 2 while having sufficient sub-sample sizes. The margin of error for this survey is 1.86%.

Self-rated mental health items asked that participants be asked to rate their mental health as excellent, very, good, good, fair, and poor, and for the analysis, it was dichotomized to poor (fair, poor) or good (excellent, very good, good). Self-rated oral health status (dependent variable) asked participants to rate their oral health status as either excellent, very, good, good, fair, and poor. Unmet oral health needs (dependent variable) were a dichotomized variable and asked if the participant had unmet needs (yes/no). The information on unmet needs was collected by asking the respondents, “have you had an unmet oral health need in the last year?” The survey also collected information on several independent variables, including, participants' age, gender, race/ethnicity, household income, education, if they live in metro or non-metro areas, and health insurance coverage. Other oral health-related questions included the last visit to the dentist and if the participants had dental insurance.

Descriptive statistics were conducted to summarize the percent and frequencies for the independent variables. Data are presented by unweighted frequencies and weighted percentages. Bivariate analyses were run to examine relationships between self-rated mental health status and oral health status and unmet need by age group, income, race/ethnicity, and gender using Chi-Square tests. Multivariable logistic regression models were used for each outcome of interest, including a respondent's self-rated oral health status and having unmet oral health needs. Covariables included self-rated mental health, age group, gender, income group, race/ethnicity, education level, metro/non-metro, occurrence of last dental visit, health insurance status, and dental insurance status. All variables of interest were included in the model, and those with *p*-values of < 0.05 were considered significant. Appropriate weights were applied to all analyses. Additional analyses included examining COVID-19 effects on life changes and mental health and oral health care utilization. COVID-19 life changes were examined by asking if individual events had occurred for a respondent within the last year, and cross-tabulated with the dichotomized mental health self-assessment variable.

## Results

Table 1 describes the mental health status of the entire sample. Those with poor mental health were disproportionately affected by life changes since the COVID-19 pandemic began. About 16% of respondents with poor mental health had lost their job, compared to 11% with good mental health (*p* = 0.0007). Those with poor mental health were also 5% more likely to be worried about losing their current job (*p* = 0.0016). Compared to those with good mental health, those with poor mental health were twice (14.5 vs. 7.5%, *p* < 0.0001) as likely to have missed a mortgage payment. Since the pandemic began 5% of the respondents will poor mental health more likely to have been threatened with eviction or foreclosure (2 vs. 7%, *p* < 0.0001), and had to move at double the rate (5 vs. 10%, *p* < 0.0001).

**Table 1:** Mental health and COVID from the State of Oral Health Equity Survey 2021 (*n* = 5,296).

	**Good mental health 4,379 (81.9%)**	**Poor mental health 917 (18.1%)**	***p*** -value*
**Covid life changes**
**Job related variables**
Lost Job	461 (10.8%)	159 (16.1%)	0.0007
Started a new job did not like	112 (2.9%)	55 (6.7%)	<0.0001
Started a new job you liked	281 (6.7%)	90 (9.7%)	0.0242
Taken a new job below your education	148 (3.3%)	56 (6.6%)	0.0008
Taken an additional Job	154 (3.3%)	48 (4.0%)	0.3805
Been worried to lose your job	445 (10.8%)	152 (15.6%)	0.0016
**Home related variables**
Missed rent or mortgage	306 (7.5%)	135 (14.5%)	<0.0001
Been threatened with foreclosure	98 (2.1%)	66 (7.3%)	<0.0001
Bought a home	173 (3.8%)	30 (3.5%)	0.7186
Had to move	214 (5.1%)	95 (9.7%)	<0.0001
**Study sample characteristics**
**Gender**			0.0191
Female	2052 (50.4%)	532 (56.3%)	
Male	2327 (49.6%)	385 (43.7%)	
**Age group**			<0.0001
18–29	560 (17.5%)	231 (35.0%)	
30–44	1196 (25.0%)	307 (25.1%)	
45–59	923 (24.8%)	199 (22.5%)	
60+	1700 (32.6%)	180 (17.5%)	
**Race/ethnicity**			0.3684
Asian	97 (5.0%)	13 (4.4%)	
Black	646 (12.0%)	118 (11.6%)	
2+	115 (2.3%)	41 (3.4%)	
Hispanic	748 (16.0%)	199 (19.5%)	
Other	63 (1.4%)	13 (0.9%)	
White	2710 (63.4%)	533 (60.2%)	
**Household income**			<0.0001
Less than $30,000	1066 (24.9%)	380 (41.8%)	
$30,000–$60,000	1236 (25.9%)	262 (27.0%)	
$60,000–$100,000	1157 (25.2%)	172 (17.9%)	
$100,000 or more	920 (24.0%)	103 (13.4%)	
**Education**			<0.0001
Less than HS	196 (8.8%)	63 (14.3%)	
HS graduate or equivalent	806 (26.3%)	211 (34.9%)	
Some college/associates degree	2103 (27.3%)	459 (29.4%)	
Bachelor's degree	746 (21.8%)	131 (14.3%)	
Post graduate study	528 (15.8%)	53 (7.1%)	
**Region**			0.4633
Non-metro area	697 (16.5%)	165 (17.8%)	
Metro area	3682 (83.5%)	752 (82.2%)	
**Last dental visit**			<0.0001
Less than 6 months ago	1931 (44.9%)	300 (30.8%)	
Between 6 months to 1 year ago	885 (19.9%)	171 (18.5%)	
More than a year and less than two years ago	690 (15.3%)	162 (17.7%)	
More than 2 and less than 5 years ago	480 (11.1%)	138 (14.1%)	
5 or more years ago	352 (7.4%)	136 (16.8%)	
Never	37 (1.5%)	10 (2.1%)	
**Dental insurance status**			0.1245
Insured	3049 (70.5%)	622 (67.0%)	
Not insured	1314 (29.5%)	293 (33.0%)	
**Health insurance status**			0.0427
Insured	3998 (91.2%)	804 (88.1%)	
Not insured	368 (8.8%)	107 (11.9%)	
**Oral health status**			<0.0001
Excellent	324 (8.4%)	18 (2.7%)	
Very good	1418 (32.7%)	89 (9.5%)	
Good	1726 (39.6%)	277 (28.7%)	
Fair	683 (14.5%)	357 (41.0%)	
Poor	217 (4.6%)	174 (18.0%)	
**Unmet needs**			<0.0001
Yes	2312 (52.8%)	651 (69.2%)	
No	2067 (47.2%)	266 (30.8%)	
**Oral health symptoms treatment**			<0.0001
Went to the dentist	1019 (44.5%)	218 (30.6%)	
Went to the ED	36 (1.8%)	18 (2.8%)	
Did nothing	1249 (53.4%)	412 (66.3%)	

* *All variables are reported at weighted n (unweighted %); p-value results from Chi-square test*.

Table 1 also provides the description of the overall sample is provided. Eighteen percent of respondents rated their mental health as poor, and 82% rated it as good. Females were 4% more likely to rate their mental health as poor compared to men (20 vs. 16%, *p* = 0.0191). About 35% of respondents age 18–29 years reported poor mental while only 17% reported good mental health. Those who had poor mental health were over three times more likely to rate their oral health as poor compared to those with good mental health (18 vs. 4.6%, *p* < 0.0001). Those who had poor mental health were less likely to be insured compared to those who reported good mental health (88 vs. 91%, *p* < 0.0001). Sixty-nine percent of respondents with poor mental health reported having one or more unmet oral health needs compared to 53% of those with good mental health reporting the same (*p* < 0.0001). Of those who had an unmet oral health need, those with self-rated good mental health were much more likely to seek care. Forty-five percent of respondents with good mental health said they visited the dentist to address their symptoms; 2% said they went to the emergency department (E.D.) and 53% said they did nothing. Only 31% of respondents with poor mental health went to a dentist to address their symptoms; 3% went to the E.D., and 66% did nothing (*p* < 0.0001).

Table 2 provides the results of the logistic regression model oral health status. Respondents with self-rated good mental health had low odds of rating their oral health as “poor” (OR = 0.22; 95% CI = 0.18, 0.26). Respondents aged 45–59 years had high odds of rating their oral health as 'poor' (OR = 1.20 95% CI = 1.0, 1.4). Respondents who self -reported belonging to more than two races were more likely to rate their oral health as poor (OR = 1.33 95% CI = 0.91, 1.94).

**Table 2:** Association between Oral Health Status and Mental Health from the State of Oral Health Equity in America survey 2021.

	**Odds ratio**	**95% confidence limits**		***p*** -value*
**Self-rated mental health (ref: poor health)**				<0.0001
Poor	1.00			
Good	0.22	0.18	0.26	
**Gender**				0.1691
Male	1.00			
Female	0.91	0.80	1.0	
**Age group**				0.0002
60+	1.00			
18–29	0.73	0.59	0.89	
30–44	0.92	0.78	1.09	
45–59	1.20	1.00	1.44	
**Race/ethnicity**				0.0046
White, non-Hispanic	1.00			
2+, non-Hispanic	1.33	0.91	1.94	
Asian, non-Hispanic	0.99	0.668	1.48	
Black, non-Hispanic	0.93	0.76	1.15	
Hispanic	0.78	0.65	0.94	
Other, non-Hispanic	0.43	0.24	0.79	
**Education level**				0.0024
Less than HS	1.00			
HS graduate or equivalent	0.89	0.66	1.20	
Vocational/tech school/some college/associates	0.88	0.67	1.18	
Bachelor's degree	0.75	0.55	1.02	
Post grad study/professional degree	0.59	0.42	0.83	
**Income group**				<0.0001
Less than $30,000	1.00			
$30,000 to under $60,000 v	0.96	0.80	1.16	
$60,000 to under $100,000	0.69	0.57	0.84	
$100,000 or more	0.55	0.45	0.68	
**Metro**				0.5041
Non-metro area	1.00			
Metro area	0.94	0.79	1.12	
**Last dental visit**				<0.0001
Never	1.00			
Less than 6 months ago	0.65	0.317	1.31	
Between 6 months ago and one year ago	0.97	0.48	1.99	
More than a year and less than two years ago	1.56	0.77	3.17	
More than 2 and less than 5 years ago	2.64	1.29	5.40	
5 or more years ago	5.05	2.47	10.34	
**Health insurance status**				0.2381
Yes	1.00			
No	1.16	0.91	1.49	
**Dental insurance status**				0.1038
Yes	1.00			
No	0.87	0.74	1.03	

* *Multivariable logistic regression*.

The odds of respondents rating their oral health as “poor” decreased with increased levels of education (H.S. graduate or equivalent OR = 0.89, 95% CI = 0.66, 1.20; Vocational/tech school/some college/associates OR = 0.88, 95% CI = 0.67, 1.18; Bachelor's degree OR = 0.75, 95% CI = 0.55, 1.02; Postgrad study/professional degree OR = 0.59, 95% CI = 0.42, 0.83).

In addition, respondents who had an income of over $30,000 had lower odds of rating their oral health as “poor” and as the income increased the odds decreased ($30,000 to under $60,000, OR 0.96; $60,000 to under $100,000, OR = 0.69; $100,000 or more OR = 0.55) Respondents who had visited the dentist in the last 6 months (OR = 0.65, 95% CI = 0.31, 1.31) and between 6 months and a year (OR = 0.97, 95% CI = 0.48, 1.99) had lower odds of rating their oral health as “poor.”

Table 3 provides the results of the logistic regression model for unmet oral health needs. Respondents with self-rated good mental health had low odds of having an unmet oral health need (OR = 0.55, 95% CI = 0.45, 0.69). Those in the highest two income groups ($60,000 to under $100,000, OR = 0.0.76, 95% CI = 0.88, 1.31; $100,000 or more, OR = 0.76, 95% CI = 0.59, 0.97) had low odds of having an unmet oral health need. Respondents aged 30–44 years (OR = 1.27 95% CI = 1.05, 1.53) and 45–59 years (OR = 1.35 95% CI = 1.11, 1.65) had high odds of rating their oral health as “poor.”

**Table 3:** Association between Unmet Oral Health Needs and Mental Health from the State of Oral Health Equity in America survey 2021.

	**Odds ratios**	**95% confidence limits**		***p*** -value*
**Self-rated mental health**				<0.0001
Poor	1.00			
Good	0.55	0.45	0.69	
**Gender**				0.1658
Male	1.00			
Female	1.11	0.96	1.29	
**Age group**				0.0144
60+	1.00			
18–29	1.14	0.91	1.44	
30–44	1.27	1.05	1.53	
45–59	1.35	1.11	1.65	
**Race/ethnicity**				0.5080
White, non-Hispanic	1.00			
2+ races, non-Hispanic	0.71	0.47	1.08	
Asian, non-Hispanic	0.93	0.54	1.59	
Black, non-Hispanic	0.98	0.78	1.23	
Hispanic	0.89	0.73	1.09	
Other, non-Hispanic	0.74	0.41	1.33	
**Education level**				0.0506
Less than HS	1.00			
HS graduate or equivalent	0.93	0.66	1.31	
Vocational/tech school/some college/associates	0.99	0.72	1.36	
Bachelor's degree	0.78	0.55	1.11	
Post grad study/professional degree	0.74	0.51	1.08	
**Income group**				0.0035
Less than $30,000	1.00			
$30,000 to under $60,000	1.04	0.84	1.28	
$60,000 to under $100,000	0.76	0.61	0.94	
$100,000 or more	0.76	0.59	0.97	
**Metro**				0.4323
Non-metro area	1.00			
Metro area	1.07	0.88	1.31	
**Last dental visit**				0.0002
Never	1.00			
Less than 6 months ago	6.35	2.60	15.52	
Between 6 months ago and one year ago	6.04	2.46	14.86	
More than a year and less than two years ago	6.18	2.50	15.28	
More than 2 and less than 5 years ago	7.21	2.90	17.91	
5 or more years ago	8.59	3.44	21.41	
**Health insurance status**				0.1421
Yes	1.00			
No	1.24	0.93	1.65	
**Dental insurance status**				0.6331
Yes	1.00			
No	0.96	0.80	1.15	

* *Multivariable logistic regression*.

## Discussion

This is the first study to evaluate the relationship between oral health and mental health during the COVID-19 pandemic and contributes to a larger but still underdeveloped body of literature on the association between oral and mental health, generally. Our survey of a representative sample of U.S. households found that about 20% of respondents reported poor mental health. About 35% of younger respondents (i.e., aged 18–25 years) and 43% of females reported poor mental health. Respondents with low income and less education also reported poor mental health. Respondents who reported poor mental health were more likely to be facing financial and emotional hardships and were more likely to have had COVID-19.

Our results are consistent with national statistics. According to the National Alliance on Mental Illness and the National Institute of Mental Health, 21% of all adults report poor mental health in the U.S. annually [15]. The National Institute of Mental Health also reports that the prevalence of mental illness is higher in females compared to males and that young adults aged 18–25 years have the highest prevalence (30%) of any mental illness compared to all other age groups [15, 16]. Given that our data were collected during the COVID-19 pandemic, we used a probability-based panel and our results were consistent with national data, our results are reliable to make national level conclusions.

Poor oral health status among the respondents was associated with poor mental health, age, socioeconomic status, and not visiting the dentist at regular intervals. There is much evidence that socioeconomic status is closely associated with oral health status, irrespective of the socioeconomic measure used [17, 18]. A recent National Health and Nutrition Examination Survey study reported that age, race/ethnicity, education, and family income were significantly associated with the oral health status of Americans [18]. In this study, whites rated their oral health as poor more often compared to those from other racial groups. These results are similar to those from previous studies which have found that racial minorities perceive less need for oral health services and have lower health literacy, thus often not rating their oral health as poor [19, 20].

There is a paucity of research on the association between poor mental health and poor oral health status. However, a few studies in this area shed light on possible underlying reasons for this association. Low prioritization of oral health, low recognition of the association between poor oral and mental health by healthcare providers, and lack of alternative service models in dental settings for those with heightened fear and anxiety and/or mental health problems have been identified as some of the underlying reasons for this association [1, 2].

Unmet oral health needs of the respondents were associated with poor mental health, income, and utilization of dental care. Over 65% of the respondents who endorsed poor mental health reported doing nothing about their oral health symptoms. Evidence suggests that individuals who have experienced a mental health disorder underutilize dental services. Reasons such as stigma, shame, helplessness, low self-esteem, lack of income and health insurance, dental fear/anxiety/phobia, and restlessness in the dental waiting environment contribute to underutilization [21, 22]. In addition, individuals who have experienced a mental health disorder may have confusion and difficulty recalling instructions, fostering mistrust between dental providers and patients [21, 23]. Also, individuals with poor mental health are more likely to belong to low-socioeconomic groups, be unemployed, and have substantial comorbidities, and these factors could contribute to and exacerbate the underutilization of dental services [24]. Other essential aspects of underutilization of dental care among individuals with mental health issues are perceived need for treatment, motivation to visit the dentist, and social and physical environmental barriers such as clinic location, the flexibility of the providers, and clinic hours [25]. Apart from patient-level factors, structural, organizational, and environmental factors such as limited specialized expertise of oral health providers in working with patients with mental health problems as well as lack of interprofessional integration between dental services and general medical and mental health services can exacerbate the underutilization [25].

Our study has some limitations. The results of the survey are based on a cross-sectional survey, and all data were self-reported by the participants. Though there are numerous self-report instruments validated for the assessment of specific mental health problems, the time and space limitations of the overall survey design limited the number of mental health-related items that could be included. Thus, only a single item assessing general mental health was used. We plan to collect similar data in 2022 to keep the research continuum and work toward better understanding the relationship between mental and oral health over time. Future studies will utilize more specific validated measures because the mental health variable lacked specificity (as it did not provide diagnostic-level information). Despite these limitations, this study offers a significant contribution to the literature, highlighting the association between oral health status and poor mental health at the population level. Further research is necessary to understand the mechanisms underlying these associations and develop models of care that will promote oral health utilization by individuals with mental health problems.

## Data Availability Statement

The datasets presented in this article are not readily available because the raw dataset for this study is the property of the CareQuest Institute for Oral Health. A request needs to be submitted to the corresponding author to get access to the raw data. Requests to access the datasets should be directed to tamanna.tiwari@cuanschutz.edu.

## Ethics Statement

This study was deemed exempt by the WCG IRB. Written informed consent for participation was not required for this study in accordance with the national legislation and the institutional requirements.

## Author Contributions

TT, AK, CR, ET, and JF-H contributed to the conception and design, data analysis and interpretation, drafted, and critically revised the manuscript. Data acquisition was done by AK, ET, and JF-H. All authors gave final approval and agree to be accountable for all aspects of the work.

## Funding

CR received support from Grant K23DE028906 in preparation of the manuscript.

## Conflict of Interest

The authors declare that the research was conducted in the absence of any commercial or financial relationships that could be construed as a potential conflict of interest.

## Publisher's Note

All claims expressed in this article are solely those of the authors and do not necessarily represent those of their affiliated organizations, or those of the publisher, the editors and the reviewers. Any product that may be evaluated in this article, or claim that may be made by its manufacturer, is not guaranteed or endorsed by the publisher.
